# Clinically Excellent Use of the Electronic Health Record: Review

**DOI:** 10.2196/10426

**Published:** 2018-10-05

**Authors:** Leah Wolfe, Margaret Smith Chisolm, Fuad Bohsali

**Affiliations:** 1 Division of General Internal Medicine Johns Hopkins Bayview Medical Center Johns Hopkins University School of Medicine Baltimore, MD United States; 2 Department of Psychiatry and Behavioral Sciences School of Medicine Johns Hopkins University Baltimore, MD United States; 3 Department of Medicine School of Medicine Duke University Durham, NC United States

**Keywords:** clinical excellence, electronic health record, electronic medical record, technology, communication skills, interpersonal skills, professionalism, humanism, patient care

## Abstract

**Background:**

The transition to the electronic health record (EHR) has brought forth a rapid cultural shift in the world of medicine, presenting both new challenges as well as opportunities for improving health care. As clinicians work to adapt to the changes imposed by the EHR, identification of best practices around the clinically excellent use of the EHR is needed.

**Objective:**

Using the domains of clinical excellence previously defined by the Johns Hopkins Miller Coulson Academy of Clinical Excellence, this review aims to identify best practices around the clinically excellent use of the EHR.

**Methods:**

The authors searched the PubMed database, using keywords related to clinical excellence domains and the EHR, to capture the English-language, peer-reviewed literature published between January 1, 2000, and August 2, 2016. One author independently reviewed each article and extracted relevant data.

**Results:**

The search identified 606 titles, with the majority (393/606, 64.9%) in the domain of communication and interpersonal skills. Twenty-eight of the 606 (4.6%) titles were excluded from full-text review, primarily due to lack of availability of the full-text article. The remaining 578 full-text articles reviewed were related to clinical excellence generally (3/578, 0.5%) or the specific domains of communication and interpersonal skills (380/578, 65.7%), diagnostic acumen (31/578, 5.4%), skillful negotiation of the health care system (4/578, 0.7%), scholarly approach to clinical practice (41/578, 7.1%), professionalism and humanism (2/578, 0.4%), knowledge (97/578, 16.8%), and passion for clinical medicine (20/578, 3.5%).

**Conclusions:**

Results suggest that as familiarity and expertise are developed, clinicians are leveraging the EHR to provide clinically excellent care. Best practices identified included deliberate physical configuration of the clinical space to involve sharing the screen with patients and limiting EHR use during difficult and emotional topics. Promising horizons for the EHR include the ability to augment participation in pragmatic trials, identify adverse drug effects, correlate genomic data to clinical outcomes, and follow data-driven guidelines. Clinician and patient satisfaction with the EHR has generally improved with time, and hopefully continued clinician, and patient input will lead to a system that satisfies all.

## Introduction

Use of the electronic health record (EHR) during clinical encounters is now a standard part of contemporary medical practice. The EHR—like other medical technologies—is designed to optimize the efficiency and quality of health care delivery, and ultimately—one hopes—improve patient outcomes. However, as anyone who has ever used or seen his/her health care provider use the EHR during a clinic visit knows that use of the EHR in a way that preserves or enhances clinical excellence is challenging. The Johns Hopkins Miller-Coulson Academy of Clinical Excellence (MCACE) has previously identified the following domains of clinical excellence: (1) communication and interpersonal skills, (2) diagnostic acumen, (3) skillful negotiation of the health care system, (4) scholarly approach to clinical practice, (5) professionalism and humanism, (6) knowledge, and (7) passion for clinical medicine [1]. To identify best practices around the clinically excellent use of the EHR, the authors conducted a literature review of the MCACE domains and the EHR.

## Methods

The concepts of the clinical excellence domains and the EHR were defined using a combination of controlled vocabulary terms applicable to PubMed and keyword terms and phrases to capture the English-language, peer-reviewed literature published between January 1, 2000, and August 2, 2016 (Multimedia Appendix 1). Citations were imported into a citation management system, and duplicates were removed. The authors ensured the search strategies captured a previously published review [2] on the topic. One author (LW, FB, or MSC) independently reviewed each article and extracted relevant data. The study was submitted to the institutional review board and deemed exempt from further review.

## Results

### Overview

**Figure 1:**
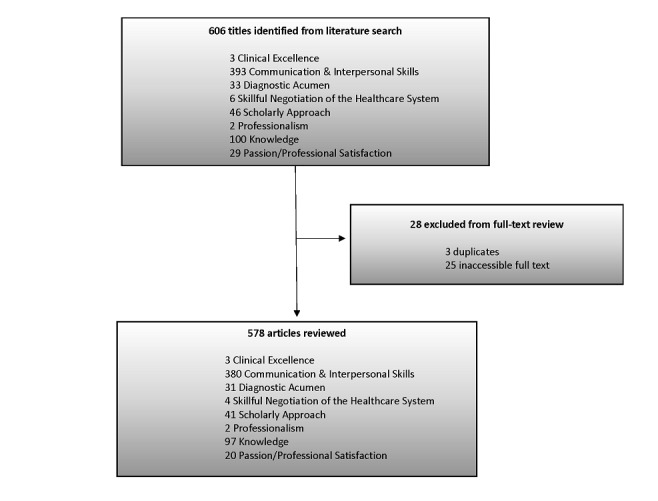
Flowchart for search strategy and review of English-language, peer-reviewed articles on clinical excellence and the electronic health record between January 1, 2000 and August 2, 2016.

The search identified 606 titles (Figure 1), the majority (393/606, 64.9%) were in the domain of communication and interpersonal skills. Twenty-eight of the 606 (4.6%) titles were excluded from full-text review, primarily due to lack of availability of the full-text article. The remaining 578 full-text articles reviewed were related to either clinical excellence generally (3/578, 0.5%) or to the specific domains of communication and interpersonal skills (380/578, 65.7%), diagnostic acumen (31/578, 5.4%), skillful negotiation of the health care system (4/578, 0.7%), scholarly approach to clinical practice (41/578, 7.1%), professionalism and humanism (2/578, 0.3%), knowledge (97/578, 16.8%), and passion for clinical medicine (20/578, 3.5%).

### Communication and Interpersonal Skills

Within the communication and interpersonal skills domain, the following practice-based themes emerged from the literature, yielding the following clinical “pearls.”

#### How Clinicians Practice

Clinicians’ baseline communication styles are the main determinants of how we communicate in the presence of EHR implying that continuing education on the basic skills of clinician-patient communication is essential as we implement the EHR [3-5]. Clinician attitudes toward the EHR can affect the attitudes of patients and the quality of clinician-patient communication in its presence [6]. It can be useful for clinicians to learn to touch type and consider the use of scribes to help optimize face-to-face communication [7-12]. Quieter keyboards can also be less disruptive to the flow of communication [2]. It is helpful for clinicians to be more transparent about their use of the EHR and to address its presence in appreciative tones [13-16].

#### Impact on Patients

Generational, cultural, and socioeconomic differences can affect patients' attitudes toward and engagement with the EHR [17]. When working with patients who speak a different language, the EHR may be both an asset and a hindrance (translation capability within the EHR can potentially mitigate this, but can be tricky) [18,19]. For example, Ratanawongsa and colleagues [19] found that increased EHR use by clinicians was associated with more biomedical statements and less positive effect from patients with low English proficiency and low health literacy. This group advocated for further research on whether the increased use of technologies like the EHR are reducing or increasing the confusion of patients with language and health literacy barriers. Studies of patient attitudes toward the EHR generally show more favorable attitudes than clinicians or researchers anticipate [5,20-23].

#### How to Prepare for a Visit

It is helpful for clinicians to review the record ahead of time to identify interval events and data, and to review the patient’s social history so that communication during the patient visit is more valuable, personalized, and less superfluous [10,12,24,25]. Clinicians can use the EHR to remind them of current life events of patients, to help personalize the visit and couch discussion of health care issues in the context of their lives [26]. Clinician-patient communication through patient portals can enhance both inter-visit and in-visit communication [27-29].

#### How to Organize the Room

Screen sharing is a significant theme in the existing literature, for the promotion of patient engagement, facilitation of communication during the visit, transparency, and patient empowerment and education. It is helpful if clinicians ensure the screen is visible to both the clinician and patient so that they share a “joint focus of attention” [2,13,25,30-36]. It is imperative that the display be large enough for the patient to view. Optimally, the room should be organized to allow eye contact between the clinician and patient at all phases of the visit [24,32,33,37-39].

#### How to Engage Patients with the Electronic Health Record in the Room

Multiple strategies can be used to improve patient engagement in visits through conscientious use of the EHR. One of these is the use of “transition phrases” or “signposting” when moving from the patient to the EHR and back [2,12,16,30,31,40-42]. It is also wise to use a language of collaboration when discussing the EHR and to address openly any issues of confidentiality [23]. It can be helpful for both patient and clinician if clinicians repeat what they write in the EHR verbally while typing—to emphasize information and messages, and to maintain a shared focus on the topic [2,40,41]. Sharing the screen with patients can facilitate communication as well—through review and verification of content, as well as through visual display of information (eg, graphics) to educate and empower [2,4,13,16,31,41,43-47]. It can even be valuable to have patients input information [12,13,48].

Clinicians should limit the use of the EHR during difficult and emotional topics [4,12,49,50], and try to maximize eye contact to avoid missing nonverbal cues and to enhance the relationship [2,4,24,25,41,51-53]. Clinicians do not want to lose the narrative and patients must have time to express their concerns, and tell their story [42,53-57]. Several studies have highlighted ways in which EHR use can facilitate provider-patient dialogue and partnership strategies, even in the context of conversations around difficult topics [2,58,50].

#### How to Use the Electronic Health Record to Enhance Intervisit Communication

Patient portals for email communication are an opportunity to enhance the flow of information and to build relationships [26,27,39,59-62]. Multiple studies exist on the use of the EHR for patient self-management of chronic disease and health behaviors [39,61,63-65]. Tasks that took time during traditional office visits can be accomplished through intervisit use of the EHR, freeing up more time for meaningful communication in the office. Direct access to test results by patients can enhance the quality and safety of care [39,61,62,66,67]. It is important to remember, however, that not all patients will have access to or identify the means of bridging that gap. The EHR has significantly increased opportunities for interprovider communication and has demonstrated benefit in transitions of care, and in the coordination of care, especially for patients with complex health needs [68-71].

### Diagnostic Acumen

Review of the literature revealed several ways in which the EHR can assist a clinician’s diagnostic acumen, such as instant access to historical records, and automation of risk score algorithms. The EHR makes access to past medical history automatic within the sphere in which the EHR operates. Retrieving outside data are the slowest area of progress, but is still improving with the EHR. The EHR’s ability to provide interconnected and immediate point-of-care access adds a new dynamic to the health care system, expanding the background of clinical knowledge and enhancing diagnostic acumen and speed of diagnosis [72].

The EHR also brings the potential to use calculated risk scores to the user’s fingertips. Physicians admitting a patient with non-ST-elevation myocardial infarction can have immediate access to the thrombolysis in myocardial infarction score. An outpatient provider can have an automated atherosclerotic cardiovascular disease risk score calculated as soon as vital signs are measured. While debate exists around the utility of these scores [73,74], they have and will continue to be ever-present in our understanding of disease. The EHR gives clinicians the added functionality of automatically calculating and providing this data as an added input to the clinician, another tool in the toolbox.

### Skillful Negotiation of the Health Care System

The EHR can help clinicians more deftly navigate the health care system to provide high-quality, cost-conscious care. One way the EHR helps clinicians improve care is by promoting adherence to guidelines. Despite knowing that guideline-directed care improves outcomes, chronic and acute-care patients receive guideline-directed care only about 50% of the time [75], and one-third of health care expenditure is wasteful [76]. Clinical decision support (CDS) is the set of prompts that highlight information that could change clinical care and is the answer to the gap in guideline-based care. CDS relies heavily on input from clinical staff who are up-to-date with guidelines. However, when done correctly, CDS has the potential to facilitate the delivery of high-quality care, improving the health of patients and avoiding unnecessary care [77,78].

Further, the Office of the National Coordinator for health care information technology is moving toward national knowledge-sharing for CDS prompts with the intent of eventually standardizing and classifying the importance of CDS. Together, these represent methods for ensuring that we are navigating our health care environment to provide succinct and concise care.

As the use of the EHR grows, data-sharing is being enhanced across networks in regional data exchange systems called health information exchanges (HIEs). With these, clinicians can share pertinent patient information, labs, and notes, as well as communicate directly about essential details. HIEs are the vehicle for creating seamless and secure data-sharing between networks.

### Scholarly Approach to Clinical Practice

Use of the EHR facilitates the creation of patient databases and undertaking of pragmatic trials [79]. Through automation of the processes of patient screening, patients can be assessed for participation in pragmatic trials directly through diagnostic codes and demographic information, and messaged at home or asked in the office if they would consent to a study. For patients with the ability to access a computer, investigators have provided informed consent via online videos which can be viewed in the comfort of the patient’s own home. Further, the addition of the computer to the clinical setting means that the networks for starting a pragmatic clinical trial are primed and ready. The data are already being collected in the system, and need only to be consented to appropriately and shared.

### Professionalism and Humanism

The human price of the EHR is the distraction. CDS popups alert clinicians to a clinical need, an incorrect allergy warning may alarm while entering a prescription, and vital signs may flag a sepsis warning inappropriately. In the rapidly advancing world of the EHR with its increased distractions, it is imperative that clinicians maintain strong bonds with patients and stop the intrusion into clinician-patient relationships [25]. Best practices described in the Communication and interpersonal skills domain can support humanistic attitudes and professional behaviors in the face of the EHR.

The electronic interface of collection is transforming the field of Patient Reported Outcomes (PROs). Many patient portals are set up to ask and record PROs, which can seamlessly integrate into the patient’s record. These PROs provide the ability to compare treatments and add patient-centered outcomes to the research. These data are being mobilized for use in decision making by groups like the Patient-Centered Outcomes Research Institute, the National Institutes of Health Collaboratory, and the American Society of Clinical Oncology.

### Knowledge

The EHR brings a new way to interface with the knowledge that clinicians generate. Two of the most exciting changes to knowledge will be the discovery of new patterns and the incorporation of genetic data to patient records via “big data” methodology. In computing, big data refers to the use of extensive datasets that are analyzed computationally to reveal previously unknown trends and associations. With enough data points, data scientists suspect that computers will eventually be able to generate prediction models for individual cases based on repositories of old case data [80]. For example, a computerized model of hyponatremia correction in newborns has been created based on large numbers of observations by computers [81].

On the forefront, data scientists and geneticists hope to incorporate patient genomic information into the EHR to help identify patterns and uncover new genetic connections. Once genetic data has been added to a patient’s profile, the EHR could theoretically learn what gene loci predispose a patient to angioedema, interstitial lung disease, or any number of previously poorly understood disease states [82,83].

In a similar vein, the EHR can automate the reporting of adverse drug reactions to newly prescribed drugs. By reporting early trends in side effects from a new agent, EHRs might accelerate the detection of untoward side effects—like myocardial infarction associated with cyclooxygenase enzyme inhibitors (ie, COX-2) [84].

### Passion and Professional Satisfaction

The introduction of the EHR was fraught with underprepared EHR platforms and unrealistic expectations. Clinicians were initially confronted with decreased efficiency, increased burnout, and high turnover. Early on, physicians using computerized order entry and electronic documentation were 30% more likely to report burnout after controlling for other variables [85]. The only intervention that routinely improved satisfaction was employing scribes, which suggests that the only positive experience associated with the EHR was minimizing its use [85]. Further analysis into trends of physician satisfaction reveals that a more robust platform is more correlated with satisfaction. Clinical notes, diagnosis function, and off-site capability were all associated with higher satisfaction. There was a trend that younger physicians were more likely to be satisfied than their elder peers [85]. Finally, and most promising of all, physicians who had access to their EHR for at least two years were 2.78 times more likely to be satisfied with their EHR compared to those with less than two years’ experience [86].

Much of the literature in other domains touched on the EHR’s potential to improve the interface with clinicians. Tools are being introduced to provide the clinician with medical references on demand for reading about developing medical data [87-89]. Finally, natural language processing is another advancing technology in which the computer attempts to interpret the clinician’s intention when writing. As an example, when a clinician diagnoses a patient with pneumonia, the EHR could ask if it should open the pneumonia order set [34,90,91]. This technology is still in its infancy and will likely require years to be ready for implementation. That said, it is one of the exciting transformations of the EHR that would produce a more fluent interface between the clinician and computer, allowing clinicians to focus back on the priority—patients.

## Discussion

Many articles published after our literature review cite the EHR as a significant factor in clinician burnout. For example, in their 2017 commentary, Shanafelt and colleagues [92] discuss clinician burnout in the era of the EHR and its attendant clerical, regulatory, and workload implications. They outline the potential broader impacts of clinician burnout for the quality of care and the health care system at large. They also emphasize the importance of measures to address the increasing documentation burden especially performance and documentation of components of care that are justifiable for billing purposes alone and do not contribute meaningfully to the episode of care. A recent systematic review by West and colleagues [93] highlights the evidence supporting both organizational and individual interventions to address burnout. Though beyond the scope of our review, clinician burnout is critical among factors that should be considered in the design, implementation, and use of the EHR going forward.

The EHR has completely transformed the clinical landscape. Its arrival and integration have been fraught with challenges, including having noticeably altered clinicians’ communication with patients. That said, clinicians are gradually transforming their approach to, and interaction with, the EHR in a way that attempts to minimize distraction and enhance the quality of the clinician-patient connection again. Computerizing this work has effectively put clinicians “on the grid” and hopefully will continue to bring positive changes to the way that clinicians gather and interact with patient data to further enhance diagnostic acumen, scholarly approach to medicine, professionalism, knowledge, passion for clinical medicine, and the ability to negotiate the health care system to provide clinically excellent care for patients.

## Appendix

Multimedia Appendix 1 PubMed search terms and process for literature review for clinically excellent use of the electronic health record.

## Abbreviations

CDS: clinical decision support
EHR: electronic health record
HIE: health information exchange
MCACE: Johns Hopkins Miller-Coulson Academy of Clinical Excellence
PROs: patient reported outcomes
