# Supplementary material for: Clinically Excellent Use of the Electronic Health Record: Review
Source: JMIR Hum Factors. 2018 Oct 5;5(4):e10426. doi: 10.2196/10426 (PMC6231887; doi:10.2196/10426)
Supplement: Multimedia Appendix 1 [file humanfactors_v5i4e10426_app1.pdf]

Appendix 1:

PubMed Search Terms and Process for Literature Review for

Clinically Excellent Use of the Electronic Health Record

Literature search was conducted in PubMed for publication dates January 1, 2001 through August 2, 2016.

“clinical excellence”[tiab] OR "excellent patient care"[tiab]

AND

"Electronic Health Records"[Mesh] OR "Electronic Health Records"[tiab] OR EHR[tiab] OR "electronic medical record"[tiab] OR EMR[tiab] OR "electronic health record"[tiab] OR "electronic documentation"[tiab]

Results: 4

"Interpersonal Relations"[Majr]) OR "interpersonal skills"[ti]

AND

"Electronic Health Records"[Mesh] OR "Electronic Health Records"[tiab] OR EHR[tiab] OR "electronic medical record"[tiab] OR EMR[tiab] OR "electronic health record"[tiab] OR "electronic documentation"[tiab]

Results: 346

("Professionalism"[Mesh]) OR "Humanism"[Mesh] OR professionalism[ti] OR humanism[ti]

AND

"Electronic Health Records"[Mesh] OR "Electronic Health Records"[tiab] OR EHR[tiab] OR "electronic medical record"[tiab] OR EMR[tiab] OR "electronic health record"[tiab] OR "electronic documentation"[tiab]

Results: 7

(("Clinical Competence"[Mesh]) AND "Diagnosis"[Mesh]) OR "diagnostic acumen"[ti]

AND

"Electronic Health Records"[Mesh] OR "Electronic Health Records"[tiab] OR EHR[tiab] OR "electronic medical record"[tiab] OR EMR[tiab] OR "electronic health record"[tiab] OR "electronic documentation"[tiab]

Results: 39

"Knowledge"[Majr] OR knowledge[ti]

AND

"Electronic Health Records"[Mesh] OR "Electronic Health Records"[tiab] OR EHR[tiab] OR "electronic medical record"[tiab] OR EMR[tiab] OR "electronic health record"[tiab] OR "electronic documentation"[tiab]

Results: 117

"Evidence-Based Medicine"[Majr] OR (scholarly[ti] AND "clinical practice"[ti])

AND

"Electronic Health Records"[Mesh] OR "Electronic Health Records"[tiab] OR EHR[tiab] OR "electronic medical record"[tiab] OR EMR[tiab] OR "electronic health record"[tiab] OR "electronic documentation"[tiab]

Results: 53

("Patient Care Management"[Majr] OR "patient care"[ti]) AND (passion*[ti] OR enthusia*[ti] OR excit*[ti] OR love[ti])

AND

"Electronic Health Records"[Mesh] OR "Electronic Health Records"[tiab] OR EHR[tiab] OR "electronic medical record"[tiab] OR EMR[tiab] OR "electronic health record"[tiab] OR "electronic documentation"[tiab]

Results: 1

(navigat*[tiab] OR negotiat*[tiab]) AND healthcare[tiab] AND system[tiab]

AND

"Electronic Health Records"[Mesh] OR "Electronic Health Records"[tiab] OR EHR[tiab] OR "electronic medical record"[tiab] OR EMR[tiab] OR "electronic health record"[tiab] OR "electronic documentation"[tiab]

Results: 6

(("Electronic Health Records"[Mesh] OR "Electronic Health Records"[tiab] OR EHR[tiab] OR "electronic medical record"[tiab] OR EMR[tiab] OR "electronic health record"[tiab] OR "electronic documentation"[tiab]))

AND

("Job Satisfaction"[Majr] OR "professional satisfaction"[ti])

Results: 28

Communication[ti]

AND

(("Electronic Health Records"[Mesh] OR "Electronic Health Records"[tiab] OR EHR[tiab] OR "electronic medical record"[tiab] OR EMR[tiab] OR "electronic health record"[tiab] OR "electronic documentation"[tiab]))

Results: 217

(("Physician-Patient Relations"[Majr] OR "doctor patient communication"[tiab]))

AND

("Electronic Health Records"[Mesh] OR "Electronic Health Records"[tiab] OR EHR[tiab] OR "electronic medical record"[tiab] OR EMR[tiab] OR "electronic health record"[tiab] OR "electronic documentation"[tiab])

Results: 190
